# Targeting microglia with lentivirus and AAV: Recent advances and remaining challenges

**DOI:** 10.1016/j.neulet.2019.134310

**Published:** 2019-08-10

**Authors:** Margaret E. Maes, Gloria Colombo, Rouven Schulz, Sandra Siegert

**Affiliations:** Institute of Science and Technology (IST) Austria, Am Campus 1, 3400 Klosterneuburg, Austria

**Keywords:** Microglia, Lentivirus, Adeno-associated virus (AAV), *In-vivo* transduction, Brain

## Abstract

Microglia have emerged as a critical component of neurodegenerative diseases. Genetic manipulation of microglia can elucidate their functional impact in disease. In neuroscience, recombinant viruses such as lentiviruses and adeno-associated viruses (AAVs) have been successfully used to target various cell types in the brain, although effective transduction of microglia is rare. In this review, we provide a short background of lentiviruses and AAVs, and strategies for designing recombinant viral vectors. Then, we will summarize recent literature on successful microglial transductions *in vitro* and *in vivo*, and discuss the current challenges. Finally, we provide guidelines for reporting the efficiency and specificity of viral targeting in microglia, which will enable the microglial research community to assess and improve methodologies for future studies.

## Highlights

• Background of lentiviral and adeno-associated virus vector design.
• Reviewing of literature that reported *in vivo* microglial transduction.
• Challenges to overcome low microglial transduction efficiency and specificity.
• Guidelines for reporting viral transduction in microglia.

## 1 Introduction

Microglia, the immune cells resident in the brain, have emerged as a critical component in neurodegenerative diseases such as Alzheimer’s and Parkinson’s disease [[1], [2], [3]]. They express several disease-associated genes, therefore modulating these genes will provide important biological insights. Because microglial function depends on the neuronal surrounding, such studies would ideally be performed *in vivo*. In recent decades, recombinant DNA vectors have enabled scientists to direct gene transcription in a cell type-dependent manner. Transfection reagents or electroporation methods introduce DNA *in vitro* but are difficult to translate to an *in vivo* environment. Transgenic mouse models can partially overcome this but are a less desirable option, combining high costs and time commitment with unpredictable success.

Employing viruses as messengers of genetic cargo has become the method of choice for targeting cells *in vivo* because it enables cell-specific delivery of genetic material within a short time period. The two main components of a viral-based gene delivery system are: first, the packaging elements consisting of structural proteins and enzymes to generate a virus; and, second, the transfer vector encoding the gene-of-interest driven under a cell type-specific promoter. Both vector components are transfected into a cell line, but only the gene-of-interest encoded on the transfer vector is packaged into the viral particles (virions). This distinguishes replication-incompetent virions that transduce a host cell, from a replicative wildtype virus, which infects host cells. To date, rabies, herpes simplex, adeno-, lenti-, and adeno-associated viruses (AAV) have been successfully used by the neuroscience community to visualize and optogenetically manipulate neuronal circuits *in vivo* [4,5]. For basic research, lentiviruses and AAVs have proven superior, offering efficient tissue transduction with no immediate immune response or cytotoxicity [5,6].

In this review, we explain the basic biology of lentiviruses and AAVs, and introduce strategies for generating viral vectors. We then review recent literature describing successful microglial transduction *in vitro* and *in vivo*, and outline the challenges for achieving viral efficiency and specificity in microglia. Finally, we provide a comparison between viral types and propose standardized measures for reporting microglial transduction experiments.

## 2 Lentivirus

**Fig. 1:**
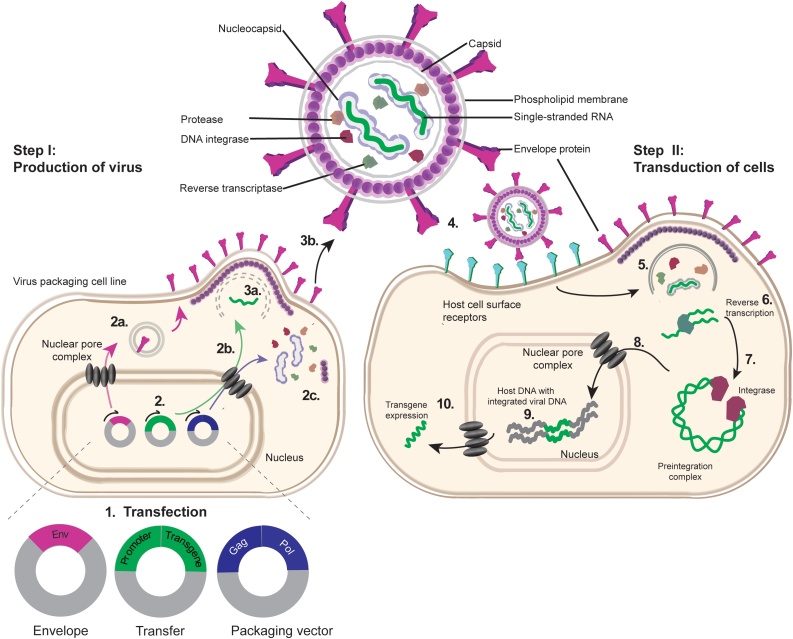
Lentiviral production (Step I) and transduction (Step II). Virus packaging cell line is transfected with envelope, transfer, and packaging vectors (1). (2) The transcribed mRNAs for the envelope, transfer, and packaging vectors are translated into: (2a) viral envelope proteins that are sorted to the cell membrane *via* the endoplasmic reticulum; (2b) single-stranded RNA viral genome; (2c) viral structural proteins and enzymes, respectively. All three components are assembled into viral particles (3a), which bud from the host cell membrane (3b). (4) Viral particles attach to the host cell surface receptors using the envelope protein. (5) Fusion of viral with host plasma membrane releases structural, enzymatic proteins, and viral core. (6) Viral RNA is reverse transcribed to double-stranded DNA that then forms a pre-integration complex with the integrase (7), which passes the nuclear pore complex (8), and catalyzes viral DNA integration into the host genome (9). (10) The transfer vector promoter drives transgene expression.

Lentiviruses belong to the *Retroviridae* family and are capable of transducing both non-dividing and dividing cells, which distinguishes them from retroviruses [[7], [8], [9]]. The genome consists of two copies of (+)-single-stranded RNA, enclosed in a capsid with structural and enzymatic proteins. These include reverse transcriptase, which converts RNA into double-stranded (ds) DNA, and the DNA integrase, responsible for integrating the dsDNA into the host genome [10]. Together, these components constitute the viral core, which is further surrounded by an envelope generated during the budding process from the host cell membrane, which harbors the viral receptors (Fig. 1).

**Fig. 2:**
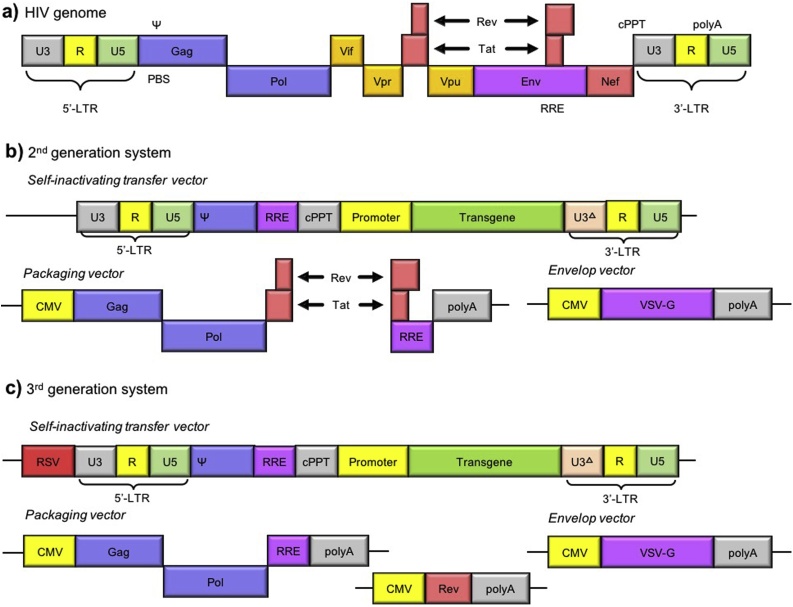
Schematic of HIV genome in (a) proviral form, and lentiviral packaging system for the 2^nd^ (b) and 3^rd^generation (c), which consists of transfer, packaging, and envelope vector. CMV: cytomegalovirus. cPPT: central polypurine tract, which initiates second DNA strand synthesis. Env: envelope protein. Gag: capsid components. HIV: human immunodeficiency virus. LTR: long terminal repeat. PBS: primer binding site for host cell tRNAs to start reverse transcription. Pol: reverse transcriptase and integrase. ψ: packaging signal for nucleocapsid assembling. R: tat-binding region. Rev: facilitates nuclear RNA genome export. RRE: rev responsive element, which serves as binding site for the viral rev protein. RSV: tat-independent transcription of viral genome. Tat: initiates transcription of the viral genome. U3: RNA polymerase II promoter for transcription of the viral genome during replication. U3^Δ^: mutated U3. Vif, Vpr, Vpu, Nef: virulence factors. Double arrows indicate splicing events.

Human immunodeficiency virus type-1 (HIV-1) is the most prominent lentivirus whose genome is well described (Fig. 2a). Its genes *gag*, *pol*, and *env*, encode structural core proteins, reverse transcriptase and integrase, and envelope glycoproteins, respectively. *Tat* and *rev* are involved in viral replication: *tat* initiates viral genome transcription and is driven by the long terminal repeats; *rev* facilitates the nuclear export of the viral mRNA and is regulated by the *rev*-responsive element. The packaging signal (Ψ) is critical for incorporating the viral genome into the capsid. Additionally, the HIV-1 genome contains virulence factors *vif*, *vpr*, *vpu*, and *nef* that interfere with the host defense mechanism and are included in the assembled virion [10].

### 2.1 Design and production of lentiviral vectors

The HIV-1 genome serves as a starting point for assembling a virion containing the transfer vector. The first generation of lentiviral vectors encoded all the packaging elements on a single vector, which posed a high biosafety risk [7]. Removing the virulence factors and separating the envelope from the packaging vector improved safety and enhanced viral replication without interfering with the production of functional HIV-1-based virions (Fig. 2b) [11]. Moreover, the 3’LTR contained a U3 deletion to abolish promoter activity for viral genome replication, and to self-inactivate the vector without affecting the virion titer or transgene expression [[12], [13], [14]]. The third, and currently preferred, generation singled out *rev* from the packaging vector, and replaced *tat* with a constitutive promoter upstream of the transfer vector (Fig. 2c) [15].

The viral envelope glycoproteins determine the lentiviral target site on the host membrane [10]. Instead of using the HIV-1 envelope gp160, which would only target cells expressing the CD4 receptor, HIV-1 can be pseudotyped with envelope glycoproteins from other viral species such as the rabies or vesicular stomatitis viruses. This increases cellular tropism and virus stability [16]. Commonly, the HIV-1 viral core is pseudotyped with the vesicular stomatitis virus glycoprotein (VSV-G), which binds to the widely expressed low-density lipoprotein (LDL) receptor [17,18].

### 2.2 Lentiviral transduction of microglia

The first attempts to achieve lentiviral transduction of microglia were in primary microglial cultures [[19], [20], [21], [22]]. Balcaitis et al. generated an HIV-1/VSV-G with a transfer vector encoding for GFP under the murine stem cell virus promoter [20]. Within 72 h after applying the virus to a mouse microglial culture, the authors found improved transduction as the ratio of virus to target cells (MOI) increased. Jiang et al. reported similar transduction efficiencies of 25% with MOI = 2.5, 55% with MOI = 5, 10, or 20, and 99% with MOI = 50 or 100 in rat microglia [21]. Microglial morphology was indistinguishable between control and MOI = 5, but MOI > 50 resulted in apoptosis starting from day 4.

The first *in vivo* lentiviral microglial transduction study was by Åkerblom et al., who transduced microglia in the rat striatum using self-inactivating-HIV-1 VSV-G-pseudotyped virions (SIN-HIV-1/VSV-G) [23]. They designed a transfer vector that encoded GFP under the ubiquitous phosphoglycerate kinase (PGK) promoter (PGK-GFP). To increase microglial specificity, the authors took advantage of an observation that there is no microRNA-9 expression in microglia, but it is commonly found in other cell types. By introducing four tandem-repeats of the microRNA-9 binding site (4miR.T) in the 3’UTR of GFP, the endogenous microRNA-9 sequesters GFP mRNA in all cells except microglia. Indeed, the authors observed 75% of Iba^+^-microglia to be GFP^+^; however, the transduction efficiency was not quantified, and there was no field-of-view image of the striatum to support the high transduction rate reported [23].

In a follow-up study, Brawek et al. performed calcium imaging of microglia in the mouse somatosensory cortex [24]. The authors used the previously described SIN-HIV-1/VSV-G but substituted the cytomegalovirus (CMV) for the PGK promoter and the calcium sensor Twitch-2B (CMV-Twitch-2B-4miR.T) for GFP. The transduction efficiency of Iba1^+^-microglia was 37% close to the injection site and 63% in the periphery. The authors also reported transduction of neurons and astrocytes near the epicenter and suspect that the endogenous microRNA-9 pool was insufficient to counteract the strong transgene transcription by the CMV promoter.

The latest study, by Nie et al., focused on how the toll-like receptors (TLR) 2/4 in microglia of the prefrontal cortex contribute to social avoidance behavior induced by repeated social defeat stress [25]. The authors generated a transfer vector based on the PGK-GFP-4miR.T construct and replaced the GFP with a double-floxed inverted open reading frame (DIO) encoding mCherry and RNAi for TLR2 and TLR4 (PGK-DIO-mCherry-TLR2/4RNAi). Upon *Cre*-mediated recombination of the DIO, mCherry and RNAi are expressed. The authors injected the virus into the Cx3cr1^tm2.1(cre/ERT2)Litt^/WganJ mouse model [26], which resulted in microglia-specific YFP and transgene expression after injection with tamoxifen [27]. After four weeks, microglial transduction efficiency was 25% and 45% for control and RNAi, respectively, and up to 90% specific for microglia. The discrepancy between efficiencies in control and RNAi transduced mice was not further addressed. This study shows that microglial specificity can be increased by combining Cre-mouse models with viral strategies.

In summary, all lentiviral *in vivo* studies thus far have taken advantage of the SIN-HIV-1/VSV-G system and adapted the promoter or transgene cargo (Table 1).

**Table 1:** Summary of studies using lentiviruses to transduce microglia *in vivo*.

LV generation	Virus coating	Promoter	Transgene	Injection site/ brain region	Species/ age	Transduction efficiency	Specificity	Validation method	Reference
3^rd^ generation SIN	VSV-G	PGK	GFP and microRNA-9 sponge	Striatum	Rat; adult	nd	75% microglia, some neurons	Iba1 colocalization	Akerblom et al. [23]
3^rd^ generation SIN	VSV-G	CMV	Twitch-2B and microRNA-9 sponge	Somatosensory cortex	Mice; 2-6 months	4-20% (% of Iba1+ cells)	Microglia, neurons, astrocytes; injection center: 36% microglia, periphery: 62% microglia	CD11b, Iba1 colocalization	Brawek et al. [24]
3^rd^ generation SIN	VSV-G	PGK	DIO mCherry and microRNA targeting Tlr2 and Tlr4	Medial prefrontal cortex	Mice; 6-12 weeks	20% control vector; 40% TLR microRNA vector (% of EYFP + cells)	80-90% microglia; some CD45+ monocytes	EYFP colocalization from Cx3Cr1CreERT2-YFP reporter mouse	Nie et al. [25]

CD11b: Integrin α-M. CD45: protein tyrosine phosphatase, receptor type C. CMV: cytomegalovirus. DIO: double-floxed inverted orientation. EYFP: enhance yellow fluorescent protein. GFP: green fluorescent protein. Iba1: allograft inflammatory factor 1. LV: lentivirus. nd: not determined. PGK: phosphoglycerate kinase. SIN: self-inactivating. Tlr2/Tlr4: toll-like receptor 2/4. VSV-G: vesicular stomatitis virus glycoprotein. YFP: yellow fluorescent protein.

## 3 Adeno-associated virus (AAV)

**Fig. 3:**
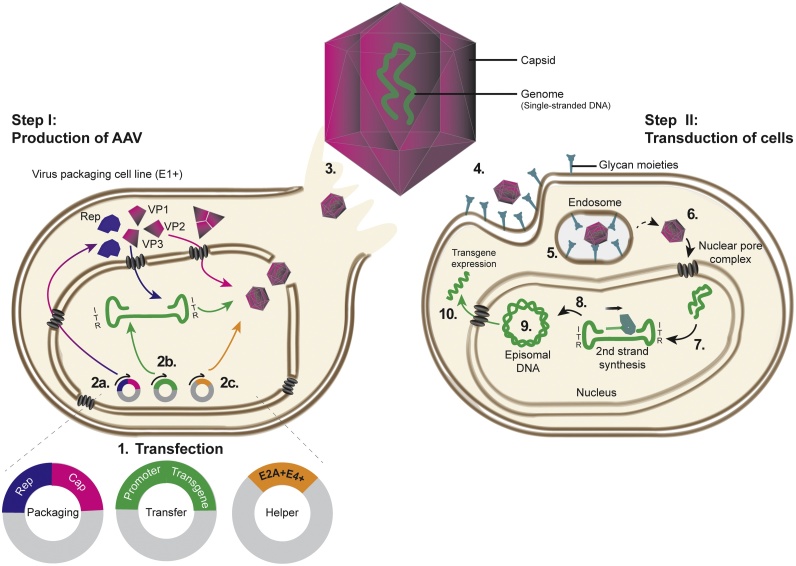
Adeno-associated virus (AAV) production (Step I) and transduction (Step II). (1) Virus packaging cell line expressing the adenovirus *E1+* is transfected with packaging, transfer, and helper vectors. (2) Transcribed mRNA from packaging, transfer, and helper vectors are translated into (2a) viral capsid (VP1, 2, 3) and Rep, which replicates the viral genome from the ITR (2b). (2c) The helper proteins support final virus assembling. (3) Viral particles are released into the supernatant. (4) Viral particles attach through interaction of the capsid with host glycan moieties, which triggers endocytosis of the particle (5). AAVs escape to the endosome by an unknown mechanism (6), and enter the nucleus *via* the nuclear core complex. (7) The viral genome is released from the capsid, the double-stranded genome synthesized (8), and an episomal circular DNA is formed (9). (10) The promoter of the transfer vector drives the expression of the transgene. ITR: inverted terminal repeats.

AAVs are part of the *Parvoviridae* family, and are small, non-enveloped viruses [28,29]. They were first identified as a contaminant of adenovirus-infected simian kidney cell culture [30]. Originally thought to be a defective virus, AAVs establish a latent infection and maintain an extra-chromosomal genome, where they can replicate autonomously (Fig. 3) [31].

**Fig. 4:**
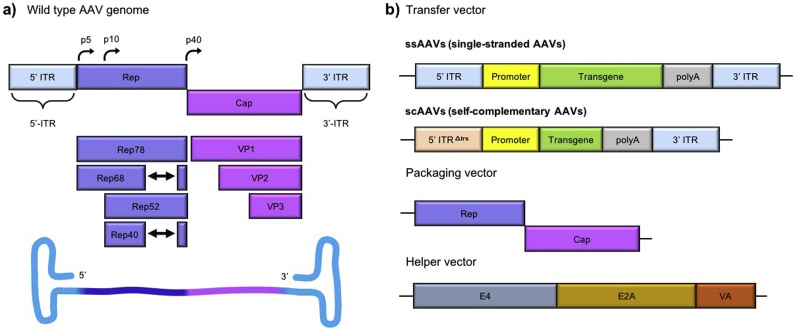
Schematic of (a) wildtype AAV genome and (b) AAV viral vectors system consisting of transfer, packaging, and helper vectors. AAV: adeno-associated virus. Cap: open reading frames for proteins (VP1, VP2, VP3) which assemble into a capsid protein shell. E4, E2A, and VA: adenoviral genes necessary for AAV lifecycle. ITR: inverted terminal repeats, which form hairpin structures. ITR^Δtrs^: on self-complementary genome, prevents rep-mediated nicking resulting in double-stranded viral genome. p5, p10, p40: promoter sequences to initiate transcription. Rep: open reading frames for proteins involved in genome replication (rep78, rep68) and packing into viral particles (rep52, rep40). trs: terminal resolution site. Double arrows indicate splicing events.

The wildtype AAV genome consists of a single-stranded DNA: *rep* encodes for proteins involved in genome replication (rep78, rep68) and virus packaging (rep52, rep40), and *cap* provides the viral capsid proteins VP1-3 [32]. Inverted terminal repeats (ITRs) flank the genome and provide the origin of replication (Fig. 4**a**) [29,33,34]. For viral replication, AAV depends on a helper virus like adeno- or herpes simplex virus that encodes for enzymes critical for the lifecycle.

### 3.1 Design and production of AAV vectors

An estimated twelve naturally occurring AAV serotypes have been identified, of which AAV serotype 2 (AAV2) is commonly used in research [[35], [36], [37]]. To generate a recombinant AAV, a packaging cell line expressing the adenoviral gene *E1+* must be transfected with a packaging vector, which supplies the *rep* and *cap* genes, a transfer vector containing the transgene flanked by ITRs, and a helper vector encoding for *E2a*, *E4*, and *VA* (Fig. 4**b**). Importantly, the transgene size is limited to 4.5 kb because the AAV capsid is only 20 nm in diameter, which constrains the size of the genome [38]. AAV pseudotyping increases tropism and efficiency: For example, AAV2/10 contains the ITR and *rep* from AAV2 and *cap* from AAV10 [[39], [40], [41], [42], [43], [44], [45], [46]].

The host cell replication machinery is required to synthesize the complementary DNA strand. This is a rate-limiting step in the AAV transduction process. Self-complementary AAVs (scAAVs) were developed to overcome this, although it further reduces the packaging capacity to approximately 2.2 kb, making it challenging to package a cell type-specific promoter and a transgene-of-interest [47,48].

### 3.2 AAV transduction of microglia

Bartlett et al. were the first to propose that AAVs are a viable option for targeting microglia *in vivo*. They injected Cy3-labeled AAV2/2 driving GFP expression under the CMV promoter into the inferior colliculus and hippocampus. Cy3 was detectable in microglia after 24 h, but microglia never expressed GFP, suggesting that the virus was either degraded or prevented from expressing the transgene [49].

In 2003, Cucchiarini et al. sought to compare AAV2/2 and AAV2/5 transgene expression levels of RFP driven by promoters from the macrophage lineages. In primary rat microglial culture, the authors describe efficiencies of 25% for F4/80, 10% for CD68, and only one cell per field-of-view for CD11b. They then injected AAV2/5-F4/80-RFP into the rat striatum and observed F4/80-RFP^+^-microglia cells, along with other unidentified cell types [50].

To find a more effective serotype, Su et al. generated several pseudotyped AAVs using a CMV-GFP transfer, and packaging vectors with capsids 2, 5, 6, 8, and 9. In primary murine microglial cultures, AAV2/6 was the most effective for transducing microglia *in vitro* as reported by the greatest fold change in GFP mRNA [19].

Rosario et al. modified the AAV6 capsid (AAV6^™^) through site-directed mutagenesis of two tyrosine residues to phenylalanine and a threonine to valine (Y731F/ Y705F/ T492V) [51]. These modifications prevent proteasomal degradation when AAV escapes the endosomal compartment [[52], [53], [54], [55]], and has been shown to increase transduction efficiency in monocyte-derived dendritic cells [[52], [53], [54]]. The authors report a consistent 95% transduction efficiency for the scAAV2/6^™^-F4/80-GFP and scAAV2/6^™^-CD68-GFP in primary microglia or mixed glial cultures, using quantitative real-time PCR. Iba^+^/GFP^+^ microglia were detectable with AAV2/6^™^ injected either into the ventricle of P0 pups or into the adult hippocampus. The authors quantified specificity, and emphasize in the text the exclusive labeling of microglia. However, the graph contradicts this statement, indicating a specificity of only 75% for F4/80 and 20% for CD68. In addition, they report a high efficiency *in vitro*, but the *in vivo* efficiency is not quantified. Overall, this study provides one of the first attempts to rationally engineer AAVs to improve microglial transduction *in vivo*.

The most recent study focused on manipulating microglia with DREADDs (Designer Receptor Exclusively Activated by a Designer Drug) to reverse pain response [56]. Grace et al. intrathecally transduced microglia in the spinal cord with AAV2/9 containing DREADD driven under the CD68 promoter. The authors had previously validated this strategy and shown that DREADDs colocalize with Iba1^+^ cells, but not with NeuN^+^ neurons or GFAP^+^ astrocytes [57]. Unfortunately, the high GFP background fluorescence levels make it difficult to evaluate this conclusion.

In summary, less than 20% AAV transduction efficiency can be achieved *in vivo* (Table 2), confirming that AAV transduction of microglia is challenging. This observation aligns with other publications that have focused on improving *in vivo* AAV transduction for other CNS cell types. Most approaches report a lack of microglial transduction upon different delivery strategies, including intrastriatal injection [44], systemic delivery [58,59], or neonatal intracerebroventricular delivery [40,60]. Only a few studies describe minimal microglial transduction without further quantification [41,61]. Although AAVs seem to be the preferred option for targeting neurons, extensive research is still needed to improve *in vivo* transduction efficiency in microglia.

**Table 2:** Summary of studies using AAVs to transduce microglia *in vivo*.

Serotype/ Pseudotype	Virus capsid	Genome	Promoter	Transgene	Injection site/ brain region	Species/ age	Validation method	Specificity	Transduction efficiency	Reference
AAV2/6	AAV6^TM^ (Y731F/ Y705F/ T492V)	sc	CBA	GFP	Intracerebroventricular (P0) Hippocampus (8wks)	Mouse; P0, 8 weeks	Iba1 colocalization	Microglia, neurons	nd	Rosario et al. [51]
			F4/80					Microglia	'modest'	
			CD68					Microglia		
AAV2/9	WT AAV9	ss	CD68	DREADD	Intrathecal	Rat; 10-12 weeks	Iba1 colocalization	Microglia	nd	Grace et al. [56,57]
AAV2/9 or AAV9*	WT AAV9	ss	CAG	GFP, ABCD1	Intravenous, Intracerebroventricular	Mouse; 8 weeks	Iba1 colocalization	Astrocytes, neurons, microglia	ICV: 3%; IV: 18%	Gong et al. [105]
AAV2/rh10	WT AAVrh10	nd	CMV	GFP	Intraspinal	Rat; adult	ED1 (CD68) colocalization	Oligodendrocytes, macrophages, microglia	20% (% of ED1+ cells)	Petrosyan, et al. [41]
AAV2/9	WT AAV9	sc	CBA	GFP	Intravenous	Mouse; P1, 8 weeks	Isolectin B_4_ and tomato lectin colocalization with GFP + cells	Neurons, astrocytes, microglia	'occasional'	Foust et al. [61]
AAV5	WT AAV5	nd	F4/80	RFP	Intrastriatal	Rat; nd	F4/80 colocalization	Microglia	nd	Cucchiarini et al. [50]

AAV: adeno-associated virus. ABCD1: ATP binding cassette subfamily D member 1. CAG: promoter consisting of CMV enhancer element, first exon and intron of chicken beta-actin gene, and the splice acceptor of rabbit beta-globin gene. CBA: chicken beta-actin promoter. CD68: cluster of differentiation 68. CMV: cytomegalovirus. DREADD: designer receptor exclusively activated by designer drugs. ED1: CD68. F4/80: EGF-like module-containing mucin-like hormone receptor-like 1. GFP: green fluorescent protein. ICV: intracerebroventricular injection. ITR: Inverted terminal repeats. IV: intravenous injection. nd: not determined. P0: postnatal day 0. RFP: red fluorescence protein. sc: self-complementary. ss: single-stranded. wt: wildtype.

* AAV genome serotype not reported.

## 4 Challenges on the path to successful microglial transduction

*In vivo* manipulation is a prerequisite to deciphering functional interaction between microglia and the neuronal environment. While viral targeting strategies have been successful in neurons, microglia remain difficult to transduce. This could be due to their endogenous macrophage function to detect, engulf, and destroy pathogens. Some viruses can circumvent this defense mechanism, for example, HIV-1 can infect microglia in the brain. To reach the nervous system, they first infect monocytes and use them as a trojan horse to pass the blood brain barrier [62]. In the following section, we point out the cellular road blocks that might prevent successful microglial transduction, and will need future investigations. This will not only be relevant for improving viral targeting of microglia, but will also provide critical biological insights into host defense mechanisms.

### 4.1 Viral entry

Viruses exhibit high affinity for defined host cell receptors, providing access to the cell upon attachment. For example, the HIV-1 envelope glycoprotein gp160 binds the CD4 receptor and co-receptors Cxcr4 and Ccr5 [63], the latter two are transcribed in microglia [64,65]. Co-receptors such as the integrins Itgav and Itgb5 [66,67], hepatocyte growth factor receptor (c-MET) [68], and fibroblast growth factor receptor 1 (FGFR1) [69,70] have been implicated in AAV attachment and internalization, from which the integrins are expressed in microglia.

Therefore, the first step towards optimizing transduction is to screen for microglial receptors, which are targeted by lentiviral envelopes or AAV capsids. This would provide the building blocks for generating pseudotyped virions. To improve attachment of the microglia-trophic AAV2/6 and 2/9, the screening must also consider membrane glycans on the microglial surface. The icosahedral AAV6 capsid binds to the glycan moieties heparin sulfate proteoglycan (HSPG) and α2-3 and α2–6 N-linked sialic acid [51,[71], [72], [73]], while AAV9 binds terminal N-linked galactose [57,74]. Such knowledge would facilitate the development of further targeted mutations of HSPG binding sites on the capsid, which has been shown to alter viral particle spread and efficiency in tissue tropism using AAV2/2 [44,75,76].

Although this strategy appears straightforward, a word of caution must be raised: microglia may elicit an unexpected response similar to macrophages. Macrophages express pattern recognition receptors (PRRs) such as toll-like receptors or sialic acid-binding immunoglobulin-type lectin 1 (Siglec1). These PRRs specialize in recognizing pathogen-associated molecular patterns (PAMPs) like viral glycoprotein structures [77]. Once PRRs recognize PAMPs, they induce a type 1-interferon response, which converts macrophages to a prophagocytic state. This leads to release of pro-inflammatory cytokines and, eventually, destruction of the virus [78]. Whether these mechanisms are also found in microglia is still under investigation, but several studies have suggested parallels [79].

Thus far, it has been shown that microglia change their receptor expression when they are in a prophagocytic state [80,81]. The current viral brain delivery systems result in local tissue damage, which leads to microglial activation [82]. On the one hand, this could be beneficial for microglial transduction since changes in the receptor distribution could enhance viral binding. On the other hand, microglia might be locally alerted to foreign viral particles and activate their internal viral defense mechanism. To circumvent this, alternative viral delivery strategies should be investigated, such as peripheral injection in the tail vein [58] or nasal administration [83].

### 4.2 Viral host defense-escape mechanisms

Once the virus successfully enters the cell, the virus must control enzymes in the host cell to ensure viral replication. Host cells, however, have developed strategies to detect and counteract a viral invasion. Lentivirus packages critical enzymes within the viral core, enabling immediate reverse transcription and integration into the genome (Fig. 1). In addition, after shedding its envelope the wildtype lentivirus releases the virulence factors Vpx and Vif, which target the host restriction factors SAMHD1 and APOBEC3 that interfere with reverse transcription and modify the reverse-transcribed viral DNA, respectively [84,85]. These virulence factors have been removed from the lentiviral vector system for biosafety reasons (Fig. 2). It would be interesting to see whether microglia express SAMHD1 and APOBEC3, and whether partially restoring the virulence factors would increase microglial transduction efficiency. Alternatively, drugs that prevent SAMHD1 or APOBEC3 activation could be developed.

Specific host defense mechanisms against AAVs have not been described. AAVs enter the cell *via* the endosomal pathway, but have to escape from the endosome before its fusion with lysosomes (Fig. 3) [[86], [87], [88]]. When and how AAVs leave the early or late endosome is still debated. Similarly, the mechanism for AAV entry into the nucleus is not known [87,89]. Only 20% of AAV2 entering the cell reaches the nucleoplasm, suggesting that several unknown mechanisms exist that prevent nuclear import [90]. Several groups have shown AAV transduction efficiency can be enhanced *in vitro* by applying drugs like bafilomycin, which prevent endosomal acidification by inhibiting the proton pump [91,92]. However, such drugs cannot be used *in vivo* because they do not cross the blood-brain barrier, and cause severe side effects [93]. Overall, a better knowledge of the host inhibitory factors, as well as the viral pathway to the nucleus, will significantly improve microglial transduction strategies.

### 4.3 Microglia-specific transgene expression

Specificity is another challenge faced when transducing microglia because often, additional cells are transduced (Table 1, Table 2). Therefore, identifying alternative constitutively expressed microglia-specific promoters is required to optimize transgene expression. Until now, the research field has focused on promoters from the macrophage lineage like CD11b, CD68, and F4/80, which also label monocytes, and show varied expression depending on the microglial activation state. For example, CD68 is more strongly expressed in proinflammatory microglia [94]. Now, with the availability of next-generation sequencing data [[95], [96], [97], [98]], it should be possible to identify genes and their corresponding promoters that are constitutively and exclusively expressed in microglia.

An alternative strategy would be to use mouse models that express Cre in a defined cell type. The transfer vector would then include the promoter and a double-floxed inverted orientation (DIO) sequence which is inverted upon Cre activation [99]. The tamoxifen-inducible Cx3cr1^CreERT2^ mouse model has frequently been used to target microglia [100]. Administering tamoxifen any time after embryonic day E14.5 induces Cre recombinase activity in microglia, blood-derived macrophages, and monocytes [101]. Due to their rapid turnover, macrophages and monocytes will be replaced by non-recombined cells within one month, leaving microglia forming more than 90% the enriched recombined population [27,99,[101], [102], [103]]. Nie et al. were one of the first to combine the mouse model and DIO-expressing lentivirus to specifically target microglia [25]. They found high specificity, suggesting that this is a valuable strategy for studying effects in adult microglia.

## 5 Lentivirus *vs* AAV – what can we learn?

In this review, we have focused on microglial transduction using lentiviruses and AAVs, each posing advantages and disadvantages for this application. Both viruses are superior to all other viral vectors (*e.g.*, adeno- or pseudorabies virus) because they have low immunogenicity and stably express transgenes without inducing cell lysis [5,6]. The most pronounced differences are in the packaging size and host genome integration. Lentiviruses can carry up to 9 kb genomic DNA, whereas AAVs are restricted to 4.5 kb, which limits the ability to use cell-specific promoters. Lentiviruses also integrate into the host genome, which results in stable transgene expression after cell division. One caveat is that this integration occurs at random, and often in regions of actively transcribed genes, which might trigger mutagenesis. In contrast, the AAV genome remains predominantly episomal and shows only infrequent quasi-random integration [104]. Both viruses have standard laboratory production protocols, although AAV is biosafety level 1, and lentivirus is biosafety level 2.

We predict that addressing a combination of the aforementioned strategies will be essential to improve efficiency and specificity of microglial transduction for any viral strategy. Based on the current literature, lentiviral approaches appear to be superior to AAVs, even though only one vector system has been implemented. A cross-wise comparison is difficult due to the variation in reporting viral methods. To improve methods for microglial transduction, and enable reproducibility, it will be important to thoroughly report all parameters for viral vector design, production, transduction methods and experimental quantification. In addition, plasmids should be shared in repositories such as Addgene to provide fast access for the entire research community. We therefore propose a guideline for best practices in reporting microglial transduction studies to increase transparency across studies and to find the most effective strategy to target microglia for manipulation.

*Guidelines for reporting viral transduction of microglia*

**Viral vector design and production**

• Which virus was used?
  ○ Generation
  ○ Serotype/pseudotype
  ○ Source of packaging and envelope vectors
• What does the transfer vector encode (promoter, regulatory elements, transgene)?
• Which viral vector production protocol was used?
• What was the viral titer?

**Viral transduction**

• *in vitro*:
  ○ Which cell line?
  ○ If primary cell culture, which protocol?
• *in vivo*:
  • Which animal model?
  • Age, sex, strain background, genotype
  • Where was the injection site?
  • What anesthesia/painkiller treatment was used?

**Determine viral efficiency and specificity**

• Report efficiency: Cells which are transgene^+^ and Iba1^+^ against total number of Iba1^+^ cells
• Report specificity: Cells which are transgene^+^ and Iba1^+^ against total number of transgene^+^ cells
• Accompanied by a field-of-view image of the brain region under investigation, including detailed field-of-view of microglial morphology.

## Competing interests

The authors declare no competing financial or non-financial interests.
